# Identification of Novel HPK1 Hit Inhibitors: From In Silico Design to In Vitro Validation

**DOI:** 10.3390/ijms26094366

**Published:** 2025-05-04

**Authors:** Israa H. Isawi, Rayan M. Obeidat, Soraya Alnabulsi, Rufaida Al Zoubi

**Affiliations:** Department of Medicinal Chemistry and Pharmacognosy, Faculty of Pharmacy, Jordan University of Science and Technology, P.O. Box 3030, Irbid 22110, Jordan; rmobeidat21@ph.just.edu.jo (R.M.O.); smalnabulsi@just.edu.jo (S.A.); rmalzoubi1@just.edu.jo (R.A.Z.)

**Keywords:** HPK1 inhibitor, Anticancer, immunotherapy, virtual screening, molecular docking, molecular dynamics

## Abstract

Hematopoietic progenitor kinase 1 (HPK1), a negative regulator of T-cells, B-cells, and dendritic cells, has gained attention in antitumor immunotherapy research over the past decade. No HPK1 inhibitor has yet reached clinical approval, largely due to selectivity and drug-like limitations. Leveraging the available structural insights into HPK1, we conducted a rational hit identification using a structure-based virtual screening of over 600,000 drug-like molecules from ASINEX and OTAVA databases. A series of molecular docking studies, in vitro kinase assays, and molecular dynamics simulations were conducted to identify viable HPK1 inhibitor hits. This approach resulted in two promising novel hit scaffolds, 4H-Pyrido[1,2-a] thieno[2,3-d] pyrimidin-4-one (ISR-05) and quinolin-2(1H)-one (ISR-03), neither of which has previously been reported as an HPK1 inhibitor. ISR-05 and ISR-03 exhibited IC_50_ values of 24.2 ± 5.07 and 43.9 ± 0.134 µM, respectively, in kinase inhibition assays. These hits constitute tractable starting points for future hit-to-lead optimization aimed at developing more effective HPK1 inhibitors for cancer therapy.

## 1. Introduction

Immunotherapy has revolutionized cancer therapy during the last decade, offering renewed hope to patients, especially those with metastatic malignancies. Unlike conventional cancer treatments that directly target cancer cells, immunotherapy boosts the patient’s immune system to more efficiently eradicate tumors [1,2,3]. The most effective approved immunotherapies are antibody-based medications that block immune checkpoints, like cytotoxic T-lymphocyte-associated protein 4 (CTLA4) and Programmed cell death protein 1 (PD1). These medications have demonstrated significant anti-tumor effects and improved patient survival; however, their therapeutic effectiveness differs depending on the type of cancer [4,5,6,7,8,9]. Due to the limited therapeutic efficacy of antibody-based medications, alternative approaches for boosting immune responses are suggested [10,11,12,13,14,15]. One effectively proposed technique involves restoring and enhancing immune response by targeting kinases that play a crucial role in regulating tumor immunity [16,17,18].

Hematopoietic-progenitor kinase-1 (HPK1), formerly known as Mitogen-activated protein kinase 1 (MAP4K1), is one of the proposed targets for modulating tumor immunity [19,20,21]. This serine/threonine kinase, highly expressed in hematopoietic cells, exerts a negative regulatory effect on the immunological responses mediated by the T-cells, B-cells, and dendritic cells. Mechanistically, HPK1 limits T-cell and B-cell receptors’ signal intensity and duration through phosphorylation/ubiquitination of the adaptor proteins SLP76 and BLNK, respectively. Studies indicate that HPK1-knockout or kinase-dead mice showed improved anti-tumor responses, evidenced by heightened T-cell activation and proliferation, increased levels of IL-2, resistance to immunosuppressive factors (like PGE_2_), and better control of tumors [21,22,23,24,25,26]. Overall, HPK1 has been shown to negatively regulate multiple phases of the cancer-immunity cycle, ranging from the initial neoantigen release and presentation to T-cell priming, trafficking, and tumor invasion [21]. Those findings demonstrated that pharmacological inhibition of HPK1 kinase activity could enhance immune cell-mediated tumor eradication, underscoring HPK1 as a compelling target for cancer immunotherapy [21,27,28].

**Figure A1:**
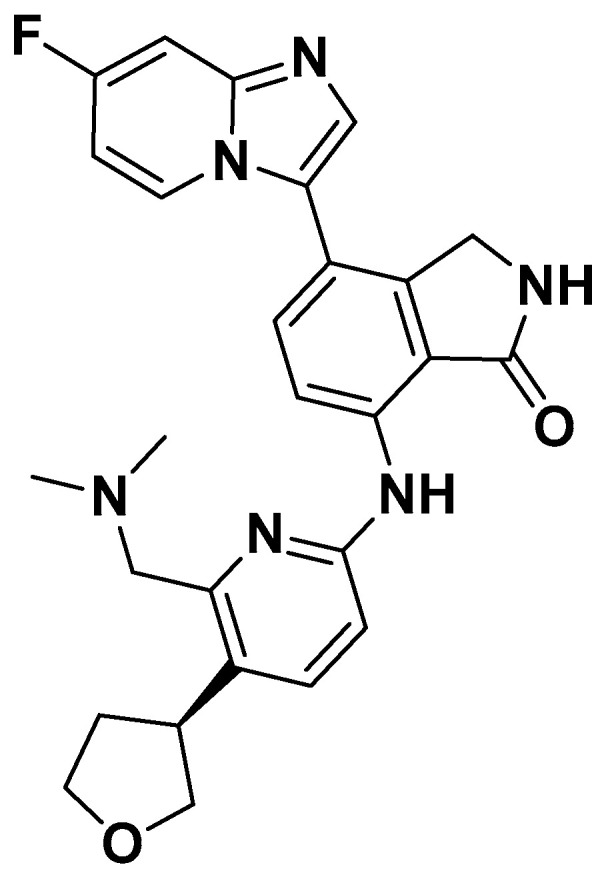
The chemical Structure of NDI-101150.

Recently, the pharmaceutical industry and academia’s discovery efforts reported several classes of ATP-competitive inhibitors of HPK1 [20,29,30]. However, most reported inhibitors in the literature have failed to overcome their selectivity and drug-like limitations, and none of them have reached the market. Meanwhile, only a few compounds progressed to clinical trials. Some examples include NDI-101150 (ClinicalTrials.gov; NCT05128487) (Figure A1, Appendix A) and CFI-402411 (Structure undisclosed) (ClinicalTrials.gov; NCT04521413), both of which are now undergoing phase I/II clinical trials “https://clinicaltrials.gov/ (accessed on 26 March 2025)”. Therefore, there is still a need to find effective and selective inhibitors for HPK1.

**Figure 1:**
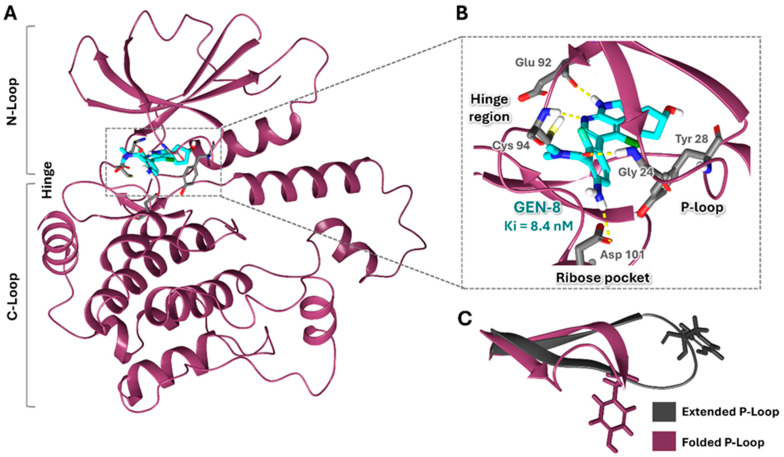
Structural Overview of the HPK1–GEN-8 Complex. (**A**) Overall structure of the HPK1–GEN-8 complex (PDB ID: 7R9T): HPK1 is depicted in dark magenta, the inhibitor GEN-8 in cyan, protein amino acid residues in gray stick representation, and hydrogen bonds as yellow dashed lines. (**B**) Close-up view of the active site highlighting key interactions between HPK1 and GEN-8. (**C**) Superimposition of extended (PDB ID: 7M0M) and folded (PDB ID: 7R9T) P-loop conformations.

One of the most promising HPK1 inhibitor scaffolds was introduced by Genentech, Inc., which identified a potent and selective spiro-azaindoline series through a high-throughput screening (HTS) campaign and structure-based drug design approaches. The co-crystal structure of GEN-8 (a spiroazaindoline derivative) and HPK1 revealed an atypical binding mode compared to other reported classes of HPK1 inhibitors (Figure 1A). GEN-8 was shown to occupy the ATP-binding site and interact with key residues in the hinge region and ribose pocket (Figure 1B). Nevertheless, the spiroazaindoline derivative was distinguished by inducing and stabilizing a unique folded P-loop conformation of HPK1, in contrast to the commonly observed extended conformation (Figure 1C) [31]. The P-loop, which typically tethers the phosphate groups of the ATP, was found to have a vital function in promoting GEN-8 inhibitory activity and selectivity. According to statistical and computational analyses in the literature, it is suggested that the formation of such a folded P-loop conformer in kinases depends essentially on the presence of aromatic residues, specifically Tyr or Phe, at position 5 of the P-loop (Tyr 28 in HPK1). This folded conformation can be induced and stabilized by inhibitors possessing a moiety that forms favorable hydrophobic interactions and/or π-π interactions with the conserved aromatic residue in that region, such as the amino-N, N-dimethylbenzamide moiety in GEN-8 (Figure 1B) [32]. The spiroazaindoline inhibitors exhibited impressive IC_50_ values and selectivity; however, their effectiveness was impeded by inadequate permeability [31].

In the present work, we employed computational methods in tandem with in vitro kinase inhibition assays to identify novel HPK1 inhibitors with promising chemical scaffolds. Leveraging HPK1’s structural knowledge, including its propensity to adopt a folded P-loop conformer that would boost inhibitor selectivity, we conducted a structure-based virtual screening (SBVS) workflow using Glide docking on nearly 600,000 lead-like compounds from ASINEX and OTAVA libraries. Fifteen compounds were selected for in vitro evaluation, which resulted in the identification of two promising hits with structurally distinct cores relative to the reported HPK1 inhibitors. Compound ISR-05 exhibited the highest potency, with an IC_50_ of 24.2 µM. Additionally, Molecular dynamics (MD) simulations were performed to provide further insights into the binding modes of the candidate hits and to assess the stability of the proposed interactions within HPK1’s active site. This study will guide future hit-to-lead optimization studies involving novel chemical scaffolds for HPK1 inhibition.

## 2. Results and Discussion

Kinases are clinically proven to be associated with more than 200 diseases, including cancer [33]. Thus, kinases represent one of the largest drug target families [34]. However, the task of developing selective kinase inhibitors is complicated by the significant structural similarities of ATP-binding sites across the kinome [35]. Off-target kinase toxicity, coupled with challenges related to the inhibitor’s pharmacokinetics, contributed to their clinical trial failures [35]. Thus, identifying more selective and safer kinase inhibitors emerges as an urgent unmet need in kinase-targeted therapeutics.

**Figure 2:**
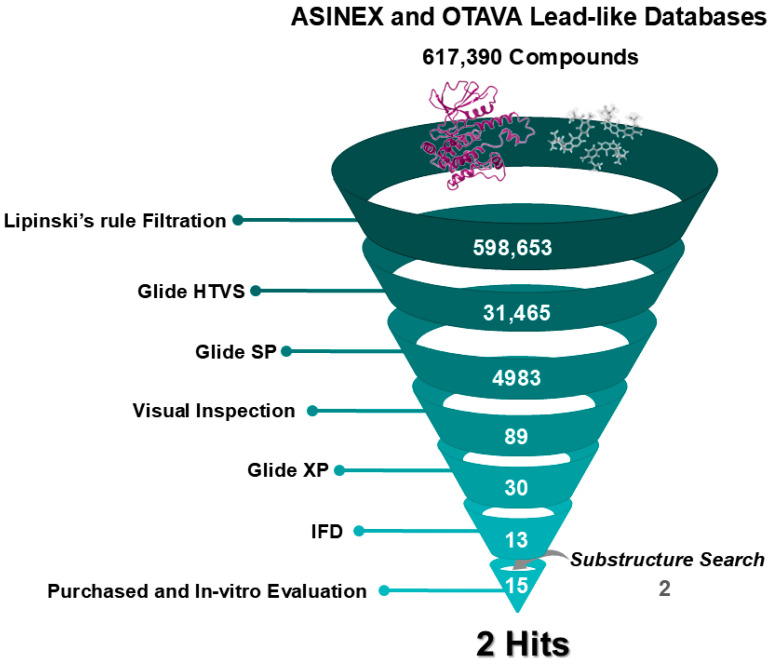
Overview of the Structure-Based Virtual Screening Workflow. A schematic representation of the screening process detailing the number of compounds retained at each filtering step.

Our target, HPK1, serves as a prime example of these challenges. Both selectivity and pharmacokinetic constraints have hindered the market entry of HPK1 inhibitors [19], highlighting the necessity of discovering more efficient and selective inhibitors. In this work, we outline our endeavors to employ SBVS via molecular docking, complemented by in vitro evaluations and MD simulations, to identify viable HPK1 inhibitor hits with distinct chemical scaffolds from those previously reported (Figure 2). Such computational screening, recognized for its time- and cost-effectiveness compared to purely experimental screening, serves as a powerful tool for accelerating hit compound discovery [36,37].

### 2.1. Structure-Based Virtual Screening

#### 2.1.1. Rationale of Structure and Database Selection

In this study, we aimed to address selectivity challenges by using the crystal structure of the HPK1 kinase domain in its folded P-loop conformation for virtual screening. This distinctive conformation, enabled by the presence of Tyr 28 in the HPK1 P-loop, distinguishes it from most other kinases that lack this capability [31,32]. We draw on the promising selectivity demonstrated by GEN-8 in targeting HPK1, which exploits this folded P-loop conformation (Figure 1) [31]. Furthermore, we postulated that the resulting smaller binding pocket of the folded P-loop would favor the identification of lower molecular weight hits. This, in turn, offers greater potential for future optimization of activity and pharmacokinetics.

To enhance chemical diversity and increase the likelihood of identifying HPK1-inhibiting compounds, two distinct chemical library databases, ASINEX and OTAVA, which collectively contained over 600,000 compounds, were chosen. In comparison to previous successful SBVS efforts that targeted an extended P-loop conformation structure (PDB ID: 6NFY; resolution of 2.17 Å) using a different library [38], our approach focuses on HPK1’s folded P-loop state.

#### 2.1.2. Filtration by Lipinski’s Rule

Prior to the SBVS, both databases were filtered according to Lipinski’s rule of five to assess drug likeness and oral pharmacokinetic suitability. This rule excludes molecules with a molecular weight above 500, a LogP value over 5, more than 5 hydrogen bond donors, or more than 10 hydrogen bond acceptors [39]. Consequently, a total of 598,653 compounds meeting these criteria were selected for screening (Figure 2).

#### 2.1.3. Validation of Docking Methods

To validate the accuracy of the docking protocols, the co-crystallized ligand from the HPK1 complex system (PDB ID: 7R9T; resolution at 2.00 Å) was extracted and re-docked into the active site using both Glide XP and IFD methodologies. The root mean squared deviation (RMSD) value for the Glide XP-docked pose was 0.91 Å, and for the top-scoring IFD pose, it was 0.44 Å relative to the original co-crystallized conformation (Figure S1A,B). As depicted in Figure S1, the docked ligands from both methods exhibited close alignment with the co-crystallized ligand, indicating that both docking protocols reliably reproduce the bound conformation, hence supporting their accuracy.

#### 2.1.4. Glide Docking

SBVS was performed using three levels of Glide docking: High Throughput Virtual Screening (HTVS), standard precision (SP), and extra precision (XP) (Figure 2). Due to the large size of the compound libraries, Glide HTVS was initially employed, yielding 31,465 compounds that advanced to the subsequent Glide SP step. Consequently, the resultant compounds were ranked based on their docking scores, which reflect an estimation of the binding free energy and incorporate the Epik state penalties (i.e., energetic cost of protonation or tautomerization of ligands). Lower docking scores suggest stronger binding affinity. Ultimately, a total of 4983 compounds were shortlisted based on moderated docking score cutoff criteria of lower than −5. These were then visually inspected for 3D interactions with critical hinge-region residues (Glu 92 and Cys 94). Eighty-nine candidates displaying suitable interactions proceeded to Glide XP where the ligand conformational sampling and scoring are more extensive than in Glide SP [40]. Afterward, a thorough examination of their XP-docked poses led to the selection of 30 high-priority hits characterized by promising binding modes, high docking scores, and key interaction residues in the active site, particularly Glu 92 and Cys 94 of the hinge, Tyr 28 and Gly 24 of the P-loop, or/and Asp 101 of the ribose pocket.

### 2.2. Induced Fit Docking and Final Selection of Hits

At the final filtering stage, the 30 compounds were subjected to Induced Fit Docking (IFD), which accounts not only for the flexibility of ligands but also for the protein, unlike standard Glide docking, leading to more accurate results. Due to the computational intensity and cost of IFD, it was reserved for this smaller subset of candidates [41]. The resulting poses were ranked according to their IFD score, reflecting both protein-ligand interaction energy and the total system energy (including ligand, protein, interaction, and complex energies), with more negative scores indicating more favorable binding. Consequently, visual inspection of binding modes and the 3D ligand-protein interactions were crucial players in the filtering process, focusing mainly on the key interactions reported from GEN-8 with HPK1 (Figure 1), such as hydrogen bonding with hinge region residues, stabilization of the folded P-loop, or/and interacting with the ribose pocket [31]. Additionally, we prioritized commercially available compounds that featured structurally diverse scaffolds relative to previously reported HPK1 inhibitors. This approach led to the selection of thirteen compounds from the SBVS for purchase and subsequent evaluation of their HPK1 inhibitory activity (Figure 2).

### 2.3. Substructure Search

During the docking analysis, certain hits stood out due to their potential for improved binding by occupying additional sub-pockets within HPK1’s active site. To explore this potential, the substructure search tool in SciFinder^n^ was used to identify extended analogs of the initial hits. These analogs were then docked into the HPK1 active site using the IFD protocol to verify their compatibility. Based on favorable docking scores, binding modes, and commercial availability, two analogs—ISR-05^a^ and ISR-15^a^ (Table 1 and Table S1)—were selected, increasing the total number of compounds advanced to testing to fifteen (Figure 2).

### 2.4. In Vitro Kinase Inhibition Assay Results

The in vitro inhibitory activity of the fifteen purchased compounds against recombinant human HPK1 was evaluated using a radiometric HotSpot^TM^ kinase assay [42]. The results were expressed as a percentage of the remaining kinase activity relative to its activity in negative control reactions (DMSO). Initial screening results at a concentration of 50 µM showed inhibitory activity for nine compounds, ranging from 3.63 to 52.39% (Table 1 and Table S1). When tested at a concentration of 25 µM, these nine compounds showed inhibitory effects ranging from 0 to 26.64%. Six hits (ISR-03, ISR-04, ISR-05, ISR-10, ISR-13, and ISR-11) were selected for IC_50_ value determination. ISR-03, ISR-05, and ISR-11 were chosen because they exhibited the highest inhibitory activity among the tested compounds, whereas ISR-04 was included for comparison with its analog ISR-05. Additionally, considering the docking studies and initial screening results, both ISR-10, which formed three H-bonds with a hinge region, and ISR-13, which exhibited interaction with Asp 101, seemed promising and were selected for IC_50_ evaluation.

**Figure 3:**
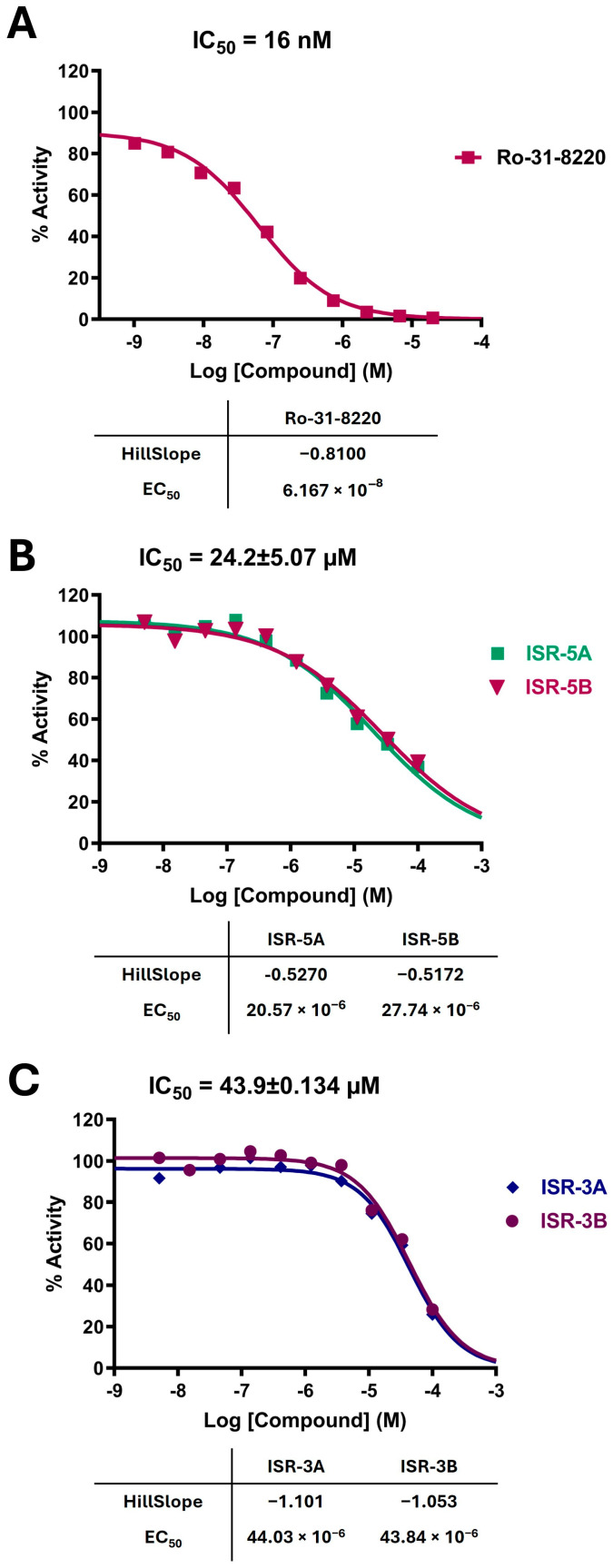
Dose-Response Curves for Positive Control, ISR-05, and ISR-03. Dose-response curves for (**A**) the positive control, (**B**) ISR-05, and (**C**) ISR-03. IC_50_ values are presented as the mean ± SD from two independent experiments.

Among these six compounds, ISR-05 and ISR-03 emerged as the most potent inhibitors, with IC_50_ values of 24.2 ± 5.07 and 43.9 ± 0.134 µM, respectively, while the other four displayed IC_50_ values above 100 µM. The dose-response curves for positive control, ISR-05, and ISR-03 compounds are shown in Figure 3A, Figure 3B, and Figure 3C, respectively.

### 2.5. Analysis of Screening Results

Throughout our SBVS workflow, complemented by a kinase inhibition assay, two HPK1 inhibitors—ISR-05 and ISR-03—were identified, each exhibiting micromolar inhibitory activity and featuring a new chemical scaffold. Their predicted binding modes, derived from IFD, are explored in detail below.

ISR-05 arose from a substructure search based on ISR-04, both of which contain a 4H-Pyrido[1,2-a] thieno[2,3-d] pyrimidin-4-one core (Table 1). The latter was originally identified by screening the OTAVA lead-like database. According to the ISR-04 IFD docking results (Figure S2), the tricyclic core interacted with the hinge region and the P-loop; however, the ribose pocket was not fully occupied. This observation promoted the idea of extending a polar moiety toward that sub-pocket, which might enhance the molecule’s activity. Consequently, a substructure search was employed to identify a derivative containing a moiety capable of forming either an additional H-bond or an ionic interaction with the ribose pocket Asp 101. Therefore, ISR-05, incorporating an additional benzyl amide, was chosen (Table 1).

**Figure 4:**
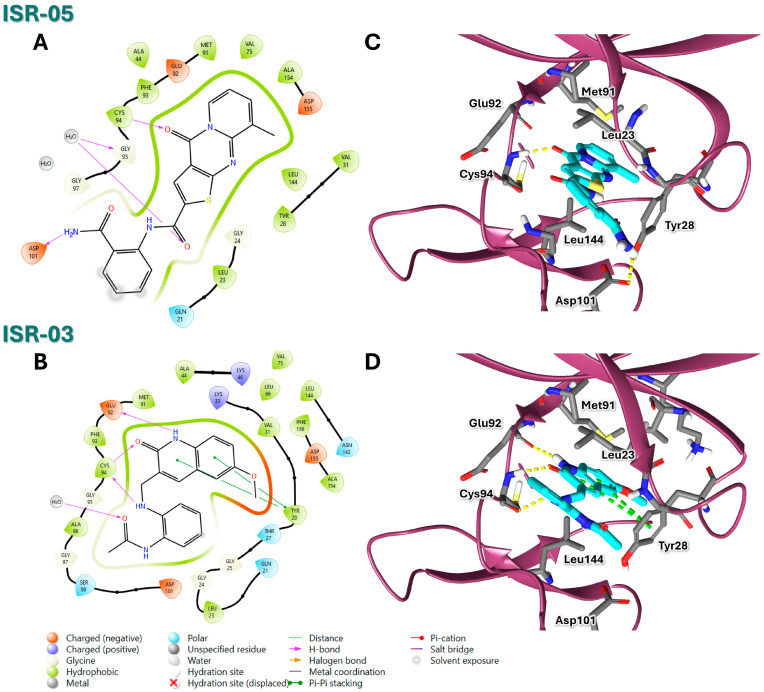
2D and 3D Representations of HPK1–Hit Interactions generated using Schrödinger, Maestro. (**A**,**B**) 2D interaction scheme of HPK1 (PDB ID: 7R9T) with ISR-05 and ISR-03, respectively. (**C**,**D**) 3D binding mode of HPK1 with ISR-05 and ISR-03, respectively. In the 3D representations, HPK1 is depicted as a dark magenta cartoon representation, inhibitors are shown in cyan stick, and amino acid carbons are represented in gray sticks. Hydrogen bonds are indicated by yellow dashed lines, while π–π stacking interactions are illustrated in green dashed lines.

ISR-05 emerged as the most active compound in this study, achieving an IC_50_ of 24.2 ± 5.07 µM (Figure 3B), whereas ISR-04 displayed an IC_50_ above 100 µM (Table 1). Analyzing their binding modes showed that both tricyclic scaffolds occupied the aliphatic hydrophobic region (Met 91, Val 31, Leu 23, Leu 144) (Figure 4A,C, and Figure S2). The carbonyl group of the pyrimidinone ring formed one H-bond with the backbone of the Cys 94 residue, anchoring ISR-05 on the hinge region, while ISR-04 formed two H-bonds with the same region. However, ISR-05 was able to establish an additional H-bond with Asp 101 in the ribose pocket by using the extended benzyl amide, a key interaction likely responsible for its superior inhibitory activity over ISR-04 (Table 1).

On the other hand, ISR-03, identified through screening the ASINEX database, showed an IC_50_ value of 43.9 ± 0.134 µM (Figure 3C). Analysis of its docking pose revealed that the quinolin-2(1H)-one core occupied the adenine pocket, surrounded and interacted with the aliphatic hydrophobic amino acids (Met 91, Val 31, Leu 23, Leu 144) (Figure 4B,D). ISR-03 also established three H-bonds with the backbones of Cys 94 and Glu 92 hinge residues. Furthermore, it formed a robust T-shaped aromatic stacking with the side chain of the Tyr 28 residue within the folded P-loop. This latter interaction likely contributes to the stabilization of the folded P-loop conformation alongside any additional hydrophobic contacts. Meanwhile, the amide group substituted on the benzene ring extended toward the solvent-exposed region.

Taken together, ISR-05 and ISR-03 with their new scaffold could be considered potential hits for further optimization toward lead compounds.

Regarding the other compounds, as illustrated in earlier sections, the predicted binding modes of compounds were included in the final selection criteria. The selected compounds formed the key interactions with active site residues needed to inhibit HPK1 effectively. In addition, their binding modes indicate the absence of any unfavorable steric clashes in the active site. Despite having promising docking scores and binding modes, not all tested compounds exhibit inhibitory activity against HPK1 as expected in the IC_50_ evaluation. Several potential factors could be the cause of this, such as solubility issues and inherent limitations in computational algorithms or scoring functions. These observations underscore the importance of validating in silico predictions with experimental assays.

### 2.6. Molecular Dynamics Simulation and Analysis

To further investigate these hits—ISR-05 and ISR-03—MD simulations were performed to assess the stability of their binding modes within HPK1’s active site under biologically relevant aqueous conditions.

**Figure 5:**
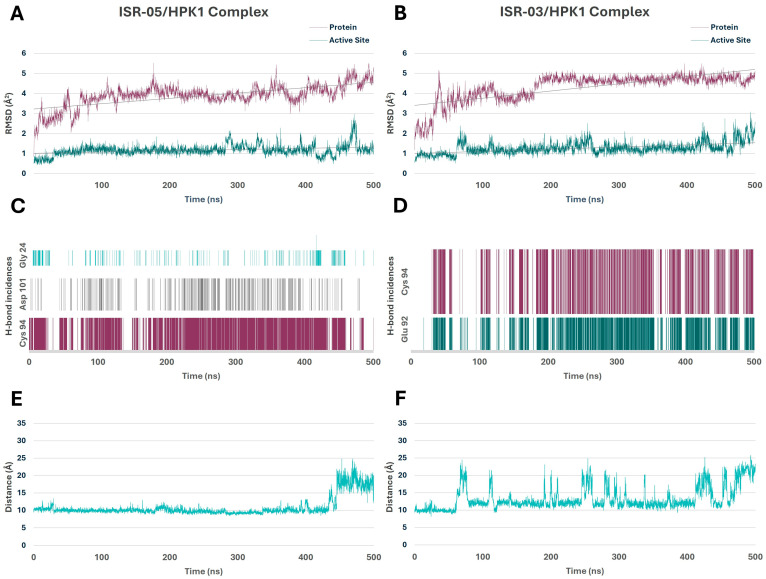
Molecular Dynamics Simulations of HPK1–Hit Complexes. (**A**,**B**) RMSD analysis of the protein backbone and active site over 500 ns for the ISR-05 and ISR-03 complexes, respectively. (**C**,**D**) Time-dependent hydrogen bond frequency analysis during the 500 ns for the ISR-05 and ISR-03 complexes, respectively. (**E**,**F**) Distance fluctuations between the Cα atom of the Cys 94 hinge residue and the CZ atom of the Tyr 28 P-loop residue during the 500 ns simulations for ISR-05 and ISR-03 complexes, respectively.

The stability of the simulation systems was evaluated by calculating the RMSD for both the backbone atoms of HPK1 and its active site over the simulation period. As shown in Figure 5A,B, the ISR-05/HPK1 and ISR-03/HPK1 complexes attained equilibrium within the first 100 ns and remained relatively stable thereafter. The protein RMSD averaged 3.88 ± 0.56 and 4.29 ± 0.69 Å^2^, while the active site RMSD averaged 1.16 ± 0.27 and 1.27 ± 0.30 Å^2^, respectively. Notably, the lower RMSD values of the active site imply that these inhibitors effectively stabilize this region.

Throughout the MD simulations, both compounds preserved their overall IFD pose in the ATP binding site and maintained the primary predicted interactions depicted in Figure 4. H-bonds—a critical factor for inhibitor binding—were tracked to assess their occupancy and stability throughout the simulation period. In the ISR-05/HPK1 system (Figure 5C), ISR-05 consistently formed a stable H-bond with the backbone of the Cys 94 hinge residue and the side chain of Asp 101 in the ribose pocket. The simulations also indicated the formation of an H-bond occasionally with P-loop residue Gly 24. Meanwhile, in the ISR03/HPK1 system (Figure 5D), two stable H-bonds were observed with the backbone of Cys 94 and Glu 92 hinge residues out of three identified in the IFD. Additional transient H-bonds were identified with the P-loop residues Thr 27 and Gly 24.Therefore, the MD results confirmed that both ISR-05 and ISR-03 form stable interactions with hinge region residues, which play critical roles in the kinase inhibitory activity. Moreover, ISR-05 engages with the ribose pocket Asp101, an interaction that proved to enhance the inhibitory potency against HPK1 of other reported inhibitor classes [29,30,31]. Thus, these hydrogen bonding interactions—whether with the hinge region or the ribose pocket—can explain the initial activity observed for our hits against HPK1.

The simulation also suggests that the interactions with Gly 24 may help stabilize the P-loop in its folded conformation. Indeed, both compounds maintain the P-loop in a folded conformation throughout the simulation. This can be demonstrated by tracing the distance between the CZ atom of Tyr 28 (in the P-loop) and another amino acid atom that would be in parallel to the Tyr while the P-loop is in the folded conformation, such as the Cα atom of the Cys 94 (in the hinge region). Distances of approximately 10 Å correlate with the folded P-loop, whereas distances of around 20 Å or more correspond to the extended conformation. Figure 5E illustrates that ISR-05 effectively stabilized the P-loop in its folded conformation over a significant portion of the simulation period (0–400 ns). Subsequently, a transition to extended conformation was observed. On the other hand, Figure 5F exhibited that P-loop has a more fluctuating conformational profile with ISR-03 with frequent transitions between the folded and extended conformations.

Overall, the MD simulation results offer insights into how ISR-05 and ISR-03 interact with HPK1, concurring in the predicted IFD binding modes. These findings offer valuable guidance for future hit-to-lead optimization efforts.

Screening efforts successfully identified two hit compounds, ISR-03 and ISR-05, that occupy the ATP binding site of HPK1. Despite these hits exhibiting micromolar activity, they possessed distinct core scaffolds compared to the potent HPK1 inhibitors in the literature. Both ISR-03 and ISR-05 hit compounds align with the reported binding mode of Type 1 inhibitors, characterized by occupancy of the adenine pocket, hydrophobic interaction with surrounding hydrophobic aliphatic residues, and anchored by H-bond interactions with key hinge region residues, as exemplified by the recently published spiro analogs series [43] and pyridine-2-carboxamide derivatives [44].

Mirroring with the structural motifs in known HPK1 inhibitors, both our hits possess solvent-extended portions, with ISR-05 specifically forming a H-bond with Asp101 in the ribose pocket, a key interaction known to enhance activity and selectivity in the diaminopyrimidine carboxamide series [45]. Moreover, akin to GEN-8 (Figure 1), both hits tend to stabilize the P-loop in its folded conformation via an H-bond with Gly 24 or stacking with Tyr28.

Tanimoto similarity analysis (Table 1) revealed lower than 0.20 similarity scores between ISR-03/ISR-05 and GEN-8 for these hits relative to GEN-8,underscoring that these hits represent novel scaffolds capable of stabilizing the P-loop through unique structural features. However, their relatively modest activity highlights the need for further optimization to establish additional interactions that could enhance binding affinity and overall potency.

### 2.7. Analyzing Pharmaceutically Relevant Predicted Properties

The QikProp tool (integrated within Maestro) was utilized to forecast many pharmaceutically relevant predicted attributes for the potential candidate hits, including aqueous solubility, brain/blood and gut/blood permeability, oral absorption, and binding to human serum albumin. These early-stage predictions can alert researchers to potential issues and guide optimization. Both compounds fall within the approved range of 95% of pharmaceuticals (Table 2). Notably, in comparison with the spiroazaindoline derivative (GEN-8), ISR-03 shows superior predicted intestinal permeability, while other predicted properties were close in value. This could highlight the promise of ISR-03, although further experimental validation is needed.

### 2.8. A Posteriori Validation of Ligand Binding Mode

To further validate our approach, we considered insights from our MD simulations, which indicated that ISR-03 and ISR-05 may interact with HPK1 when the P-loop adopts an extended conformation. To further explore this possibility, we performed both rigid Glide XP and IFD experiments using the HPK1 crystal structure with an extended P-loop conformation (PDB ID: 7R9N; resolution of 1.50 Å) [31].

Interestingly, Glide XP docking results with 7R9N produced less favorable scores for both ligands compared to our initial docking studies using the folded P-loop conformation (PDB ID: 7R9T) (Table S2). Moreover, ISR-03 adopted a different binding pose in the 7R9N active site relative to 7R9T yet retained several key interactions. Meanwhile, ISR-05 failed to reproduce any of the key binding interactions identified in our studies. Consistent with the Glide XP results, IFD docking scores were also lower for 7R9N compared to 7R9T (Table S2). While ISR-03 again exhibited a distinct binding pose, ISR-05 aligned similarly to its pose in 7R9T, albeit with reduced docking affinity.

Collectively, these consistent findings from both Glide XP and IFD docking using the 7R9N structure suggest that the structural characteristics and potential conformational state of 7R9T are essential for the effective binding of ISR-03 and ISR-05. This reinforces the validity of our initial structure-based identification strategy.

### 2.9. Limitations and Future Work

Despite the significant role of computer-aided drug design in modern drug discovery, it faces several limitations. Structure-based approaches may not fully capture the dynamic nature of proteins or accurately predict all binding interactions, even with the aid of MD simulations. Moreover, inherent constraints in docking algorithms, scoring functions, and database searches can hinder the identification of compounds that fully meet in silico criteria. To overcome these challenges, the synthesis of additional derivatives is essential for gaining a more comprehensive understanding of the structure-activity relationships (SAR) of the identified compounds.

Our future work will concentrate on the design and synthesis of more potent derivatives of ISR-05 and ISR-03. These efforts will be guided by SAR analysis and computational modeling, aiming to enhance both inhibitory potency and selectivity. Special attention will be given to incorporating functional groups that reinforce interactions with key binding site residues, and to extending the molecular scaffold toward solvent-exposed or hydrophobic regions—such as Asp101 and Asp155—to further improve HPK1 inhibition.

As this study was conducted using in silico and enzyme-based in vitro approaches, additional research is needed to validate the biological activity of these compounds and their derivatives in cell-based in vitro models. Therefore, cell-based cytotoxicity assays using relevant breast cancer cell lines, such as MCF-7 and MDA-MB-231, to evaluate their anti-cancer potential at the cellular level would be performed. In parallel with these efficacy studies, we plan to conduct preliminary ADME profiling to obtain critical insights into their potential pharmacokinetic properties of the most promising lead candidates identified through SAR analysis and initial in vitro screening. Additionally, carrying out preliminary in silico kinome-wide profiling of the optimized analogs to assess their selectivity across a panel of more than 400 kinases.

## 3. Materials and Methods

### 3.1. Preparation of Ligands

Screening sources included the two commercially available databases ASINEX (ASINEX, Amsterdam, The Netherlands) [46] and OTAVA lead-like (OTAVA Ltd., Vaughan, ON, Canda) [47]^.^ The Lipinski filtration protocol in Discovery Studio (DS) 2022 (BIOVIA Software Inc., Dassault System, San Diego, CA, USA) was used to remove compounds with physicochemical properties that were deemed inappropriate. Then, all compounds were prepared by the Ligprep module [48] (Schrödinger, Release 2024-2, New York, NY, USA) with the following parameters: energy minimization using OPLS4, generation of the possible tautomer and ionization states at pH 7.4 ± 0.2 using the classic Epik feature [49], and generating at most 32 conformers per ligand.

### 3.2. Preparation of Protein

The three-dimensional crystal structure of the HPK1 complex with a spiroazaindoline derivative inhibitor (PDB ID: 7R9T; resolution of 2.00 Å) was retrieved from the protein data bank “https://www.rcsb.org/ (1 November 2023)” [31]. The crystal structure was evaluated for missing residues using Schrodinger’s Protein Report feature, and Chain A, which includes all binding site residues, was selected over Chain B for docking studies. Subsequently, HPK1 was prepared using Protein Preparation Wizard (Schrödinger, Release 2024-2, NY, USA), following standard procedure [50]. This included adding hydrogen atoms, filling in missing side chains, and protonation state assignment using PROPKA at pH 7.4 [51]. The protein was minimized using the OPLS4 force field, and water molecules that were more than 6.00 Å away from the heteroatoms group were removed.

### 3.3. Grid Generation

To determine the position and size of the HPK1 active site, a grid was generated using the prepared chain A of HPK1 and the Receptor Grid Generation panel (Schrödinger, Release 2024-2, NY, USA). The grid center was determined based on the co-crystallized ligand, while the grid box size was set to 20 × 20 × 20 Å. A scaling factor of 0.90 was applied to the Van der Waals radii of non-polar atoms in the receptor to facilitate ligand docking, given that the protein is in rigid form during the Glide docking, and to reduce steric clashes. Moreover, due to the important role of Cys 94 and Tyr 28 residues in the interactions with known active inhibitors, the thiol and hydroxyl groups of these amino acids were designated to be treated as rotatable groups in the grid generation process.

### 3.4. Structure-Based Virtual Screening Workflow

The prepared ligands from the ASINEX and OTAVA databases were screened using a three-tier Glide-docking protocol in Schrödinger Software, Release 2024-2, (Figure 2) [52,53]. Initially, rapid high-throughput screening was carried out utilizing Glide HTVS, and the resulting hits were ranked based on their docking scores, ranging from −10.310 to 3.912. Compounds with scores greater than −8 were discarded, leaving 31,465 hits for Glide SP docking at the next level. Those with docking scores below −5, indicating favorable docking results, were visually inspected to analyze the ligand-protein interactions. Subsequently, compounds that exhibited favorable interactions, mainly with hinge amino acids, were retrieved and subjected to Glide XP docking to select potential hits. Key docking parameters include scaling the non-polar atoms of ligands by 0.90, enhancing the planarity in conjugated π systems, and penalizing non-planar amide conformations of ligands. All other parameters were left in their default settings.

### 3.5. Induced Fit Docking Study

IFD standard protocol (Schrodinger, Release 2024-2, NY, USA) was employed to generate precise ligand-protein prediction complexes, considering the flexibility of both the protein and the ligand [54]. Ligand parameters were configured to generate conformers and sample ring conformations within an energy window of 2.5 kcal/mol, penalize non-planar amide conformations, and enhance the planarity of π-conjugated groups. The ligands were initially docked using Glide, generating several ligands poses, with a Van der Waals scaling factor of 0.90 for both ligand and protein. Subsequently, the residues located within a 5.00 Å radius of the ligand poses were subjected to refinement using prime to accommodate the poses, optimizing side chains and performing a minimization step for both ligand and selected residues [55]. During the last stage of IFD, the ligands were re-docked in the induced-fit structure using Glide XP with default settings. The final IFD score reflects the energy of the optimized ligand-protein complex, shedding light on the binding affinity and complex stability.

### 3.6. In Vitro Kinase Inhibition Assay

The selected compounds were purchased from ASINEX (ASINEX, Amsterdam, The Netherlands) and OTAVA lead-like (OTAVA Ltd., Vaughan, ON, Canada) via local vendors. Then they were biologically evaluated against human recombinant active HPK1 using the radiometric HotSpot^TM^ kinase assay (the assay was performed at Reaction Biology Corp., Malvern, PA, USA [42]. Initially, the kinase inhibition percentages of the compounds were measured at two different concentrations (25.0 and 50.0 µM) in one independent experiment in duplicate mode. The compounds that showed good inhibitory activity in the initial screening had their IC_50_ values determined. Dimethyl sulfoxide (DMSO) and Ro-31-8220 (Figure 3A), which is a known ATP-competitive inhibitor for many kinases, were used in the reactions as negative and positive controls, respectively. In brief, this assay was performed by preparing the kinase substrate (myelin basic protein) with a concentration of 20 µM in freshly prepared base reaction buffer (20.0 mM Hepes (pH 7.5), 10.0 mM MgCl_2_, 1.0 mM EGTA, 0.01% Brij35, 0.02 mg/mL BSA, 0.1 mM Na_3_VO_4_, 2.0 mM DTT, and 1% DMSO). Then, the kinase enzyme was delivered into the substrate solution by gently mixing, followed by adding the tested compounds dissolved in 100% DMSO using the Echo550 liquid handler and incubated for 20 min at room temperature. Finally, the reaction was initiated by the addition of ^33^P-γ-ATP with a concentration of 10.0 µM and incubated for 2 h at room temperature. The percentage of kinase activity was detected using the P81 filter-binding method. For IC_50_ determination, the compounds’ inhibitory activity was tested at 10 concentrations (100 µM, 33.3 µM, 11.10 µM, 3.70 µM, 1.23 µM, 0.412 µM, 0.137 µM, 45.70 nM, 15.20 nM, and 5.08 nM) in one independent experiment in duplicate mode and prepared using 3-fold serial dilutions. The percentage of remaining kinase activity in tested samples was determined first, and then Prism GraphPad software version 10.2.0 (nonlinear regression/variable slope) was used to obtain the IC_50_ values and curve fits.

### 3.7. Molecular Dynamics Simulations

Molecular dynamics (MD) simulations were carried out to investigate the binding of ISR-03 and ISR-05 individually in complex with HPK1, using their IFD poses as starting structures. Simulation systems were constructed using CHARMM-GUI Solution Builder, solved in a TIP3P octahedral water box, and neutralized by the addition of 0.15 M KCl, generating Amber input files [56]. AMBER22 (University of California, San Francisco, CA, USA) was used to perform 5000 steps of minimization and 125 ps of equilibration for each system employing an NVT ensemble [57]. The next step involved conducting 500 ns of production trajectories under NPT conditions. The collected MD trajectories were visualized and analyzed in VMD (Visual Molecular Dynamics) [58].

### 3.8. Physicochemical Properties Analysis

The physicochemical properties of ISR-03 and ISR-05 were predicted using the Schrodinger suite’s QikProp tool. The predictive properties can potentially guide the future optimization and modification processes of hits [59].

## 4. Conclusions

HPK1 kinase, a cellular inhibitor of T-cells, B-cells, and dendritic cells, has emerged as a cutting-edge target for antitumor immunotherapy. Despite considerable efforts, no HPK1 inhibitor has reached clinical approval, and most reported inhibitors have been unsuccessful in overcoming their inadequate selectivity or drug-like properties. This study utilized computational methods in combination with in vitro evaluation to identify new HPK1 inhibitor hits featuring new chemical scaffolds. The SBVS utilized Glide-docking screening on two commercially available lead-like libraries (ASINEX and OTAVA), using an HPK1 with a unique folded P-loop structure. Candidate compounds were further corroborated via visual inspection and IFD.

Successfully, two hit compounds, ISR-05 and ISR-03, demonstrated micromolar inhibitory activity against HPK1, with IC_50_ values of 24.2 ± 5.07 and 43.9 ± 0.134 µM, respectively. MD simulations indicated that stable hydrogen bonds, predominantly with hinge-region residues, contribute to their inhibitory effects. These two compounds own new chemical scaffolds that have not yet been identified among HPK1 inhibitors and would provide a foundation for hit-to-lead optimization studies aimed at developing more effective HPK1 inhibitors. Planned SAR iterations and kinome-wide selectivity panels will enhance the refinement of these scaffolds into pre-clinical leads. These findings may ultimately contribute to the development of HPK1-targeted immunotherapies.

## Figures and Tables

**Table 1:** Docking scores and HPK1 inhibitory activity of the ISR-03, ISR-04, and ISR-05.

ID	Chemical Structure	Glide XP Docking Score (kcal/mol)	Induced Fit Docking Score (kcal/mol)	% Inhibition at 25 µM	% Inhibition at 50 µM	IC_50_ (µM)	Tanimoto Similarity Score ^b^ Relative to GEN-8
**ISR-03**	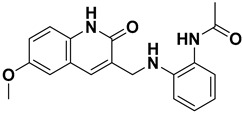	−9.70	−11.26	26.64	52.39	43.9	0.016
**ISR-04**	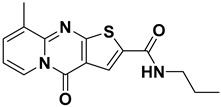	−8.44	−10.87	2.44	10.06	>100	0.018
**ISR-05** ^a^	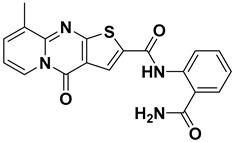	ND	−10.47	0	13.92	24.2	0.019

^a^ Hits resulted from substructure search. ^b^ Tanimoto similarity score calculated using canvas implanted in maestro.

**Table 2:** QikProp properties of the selected candidate hits.

ID	QP log S ^a^	QP log BB ^b^	QP P Caco ^c^	QPP MDCK ^d^	Percent Human Oral Absorption ^e^	QP log Khsa ^f^
ISR-03	−4.685	−0.975	656.244	313.776	95.024	0.201
ISR-05	−4.567	−1.451	150.376	91.940	77.169	−0.051
GEN-8	−4.955	−1.235	210.370	164.248	84.130	0.290

^a^ Predicted aqueous solubility; S in mol/L (−6.5 to 0.5). ^b^ Predicted log of the brain/blood partition coefficient (−3.0/1.2). ^c^ Predicted Caco-2 cell permeability in nm/s (intestinal drug permeability) (<25 is poor, >500 is excellent). ^d^ Predicated permeability across the Madin–Darby Canine Kidney (MDCK) cell monolayer (intestinal drug permeability) (<25 nm/s is poor, 25–500 nm/s is moderate, >100 nm/s is high). ^e^ Percentage of human oral absorption in GI (<25% is poor, >80% is high); (range of 95% of drugs). ^f^ Predicated the binding to human serum albumin (Acceptable range −1.5 to 1.2).

## Data Availability

The authors confirm that the data supporting the findings of this study are available within the article and its Supplementary Materials.

## Supplementary Materials

The following supporting information can be downloaded at: https://www.mdpi.com/article/10.3390/ijms26094366/s1.

## Abbreviations

**Abbreviation**	**Definition**
Asp	Aspartic acid
ATP	Adenosine Triphosphate
BLNK	B-cell Linker Protein
CTLA4	Cytotoxic T-Lymphocyte-Associated protein 4
Cys	Cystine
DMSO	Dimethylsulfoxide
DS	Discovery Studio
DTT	dithiothreitol
EGTA	Ethylene Glycol-bis(β-aminoethyl ether)-N,N,N’,N’-Tetraacetic Acid
Glu	Glutamic acid
Gly	Glycine
HPK1	Hematopoietic Progenitor Kinase 1
HTS	High Throughput Screening
HTVS	High Throughput Virtual Screening
IC_50_	Half-maximal inhibitory concentration
IFD	Induced Fit Docking
IL-2	Interleukin-2
Leu	Leucine
MAP4K1	Mitogen-Activated Protein Kinase Kinase Kinase Kinase 1
MD	Molecular Dynamics
Met	Methionine
PD1	Programmed Cell Death Protein 1
PDB	Protein Data Base
PGE_2_	Prostaglandin E2
Phe	Phenylalanine
RMSD	Root Mean Square Deviation
SAR	Structure-Activity Relationship
SBVS	Structure-based Virtual Screening
SLP76	SH2 domain containing Leukocyte Protein of 76kDa
SP	Standard Precision
TIP3P	Transferable Intermolecular Potential With 3 Points
Tyr	Tyrosine
Val	Valine
VMD	Visual Molecular Dynamic
XP	Extra Precision
