# Supplementary material for: Identification of Novel HPK1 Hit Inhibitors: From In Silico Design to In Vitro Validation
Source: Int J Mol Sci. 2025 May 4;26(9):4366. doi: 10.3390/ijms26094366 (PMC12072202; doi:10.3390/ijms26094366)
Supplement: Supplementary file 1 [file ijms-26-04366-s001.zip › ijms-3602929-supplementary.pdf]

Supplementary Material

Identification of Novel HPK1 Hit Inhibitors: From In Silico Design to In Vitro Validation

Israa H. Isawi *, Rayan M. Obeidat, Soraya Alnabulsi and Rufaida Al Zoubi

Department of Medicinal Chemistry and Pharmacognosy, Faculty of Pharmacy, Jordan University of Science and Technology, P.O. Box 3030, Irbid 22110, Jordan; rmobeidat21@ph.just.edu.jo (R.M.O.); smalnabulsi@just.edu.jo (S.A.); rmalzoubi1@just.edu.jo (R.A.Z.)

* Correspondence: ihisawi@just.edu.jo; Tel.: +962-791162595

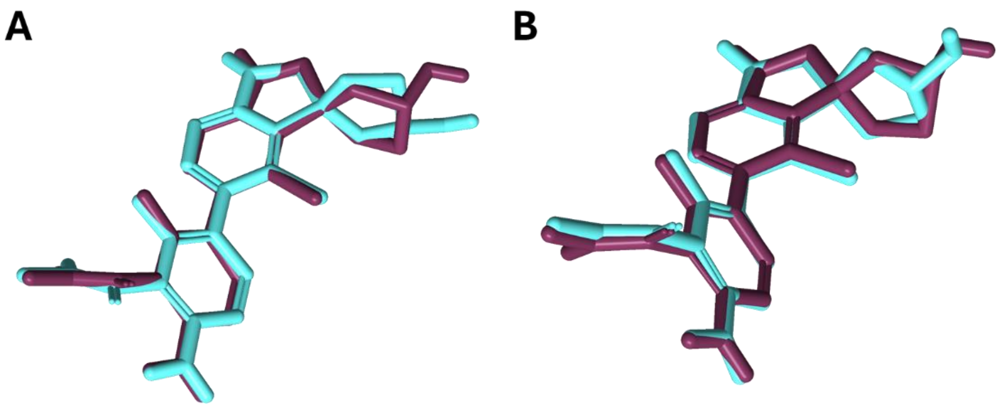

Figure S1. Superimposition of Docked and Crystallized Poses. Docked pose (A) from Glide XP and (B) from IFD (cyan) overlaid on the original crystallized pose (dark magenta).

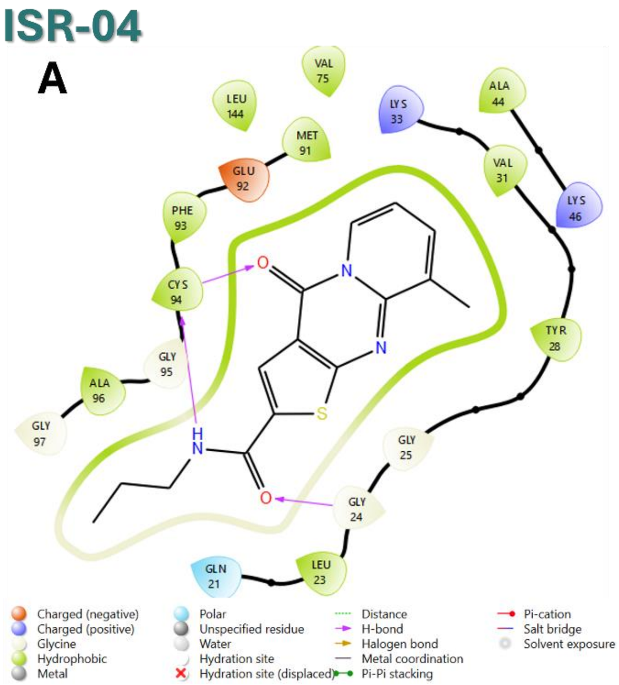

Figure S2: 2D and 3D Representations of HPK1–ISR-04 Interactions. (A-B) 2D interaction scheme of HPK1 (PDB ID: 7R9T) with ISR-04. (B) 3D binding mode of HPK1 with ISR-04 2D interaction schemes generated using Schrödinger Maestro. HPK1 is depicted as a dark magenta cartoon, inhibitors are shown in cyan stick representations, amino acid carbons are represented in gray sticks, and hydrogen bonds are indicated by yellow dashed lines.
